# *RAB37* multiple alleles, transcription activation and evolution in mammals

**DOI:** 10.7150/ijbs.47959

**Published:** 2020-09-19

**Authors:** Xu Xu, Mengxin Hu, Ruhong Ying, Juan Zou, Lan Lin, Hanhua Cheng, Rongjia Zhou

**Affiliations:** Hubei Key Laboratory of Cell Homeostasis, College of Life Sciences, Wuhan University, Wuhan 430072, China.

**Keywords:** transcription factors, genomic variation, RAB37, SNPs, mammals

## Abstract

Detecting selection signatures in genomes that relates to transcription regulation has been challenges in genetic analysis. Here, we report a set of transcription factors EBF1, E2F1 and EGR2 for transcription activation of *RAB37* promoter by a comparative analysis of promoter activities of *RAB37* in humans, mice, and pigs. Two of the transcription factors bound to and co-regulated *RAB37* promoter in each species. SNPs were further screened in pig *RAB37* gene by population genomics in pig populations from both China and Europe. Three SNPs were identified in second CpG island upstream of core promoter of *RAB37*. These SNP variations led to at least 5 haplotypes, representing 5 multiple alleles of* RAB37* in pig population. Distribution of these alleles in different genetic background of breeds showed a role of artificial selection for the variations of these multiple alleles. Of them, *RAB37-c* acquired the highest ability to activate gene expression in comparison with the other promoters, thus enhanced autophagy efficiently. These findings provide better understanding of transcription activation of *RAB37* and artificial selection via *RAB37* for autophagy regulation.

## Introduction

Macroautophagy (hereafter autophagy) is an evolutionarily conserved process of catabolism, in which intracellular unwanted components and organelles are sequestered by double-membrane vesicle and delivered to the lysosome for degradation and recycling. Autophagy pathways usually degrade cytoplasmic components, and yet selective nuclear autophagy (nucleophagy) has been observed in eukaryotic cells 1. Thus, autophagy controls both cytoplasmic and nuclear degradation, which are essential for cell survival, tissue homeostasis and organ development. Accordingly, dysregulation of autophagy is associated with a large variety of diseases, including metabolic disorders, neurodegeneration, and cancer 2.

Autophagy occurs when cells encounter different forms of stress in various physiological and pathological conditions, including nutrient deprivation and hypoxia. A range of signaling activated phagophore induction process, which were involved in a set of ATGs proteins 3. Upon starvation induction, the ULK1/ATG1 kinase complex was first activated, which consisted of ULK1, ULK2, ATG13, RBCC1/FIP200, and ATG101. The PtdIns3K kinase complex was then activated, including PIK3C3/VPS34, PIK3R4/VPS15, BECN1, ATG14, and UVRAG) 4. For phagophore expansion, two ubiquitin-like conjugation complexes had essential roles 5. ATG5 conjugated to ATG12 and interacted with ATG16L1 to form the ATG5-ATG12-ATG16L1 complex via the action of ATG7 and ATG10. The LC3B/ATG8 conjugated with ATG3 via the action of ATG4 and ATG7 sequentially. Finally, conjugation of LC3B to PE (phosphatidylethanolamine) formed a lipidated LC3B-PE and promoted autophagosome expansion. The ATG5-ATG12-ATG16L1 complex plays a key role in the process of LC3B lipidation 6. Autophagosome was then fused with lysosome to form autolysosome, and sequestered substances were degraded by lysosomal enzymes and recycled for cell reuse. Although, extensive studies detail molecular mechanisms in regulation of autophagosome formation under various physiological and pathological conditions, in particular, remains to be further explored.

RAB37, as a small GTPase, plays important roles in several cellular processes through intracellular membrane traffic. RAB37 can regulate insulin secretion in β-cells 7 and TNF-α secretion from activated macrophages 8. In allergic responses, RAB37 regulated mast cell degranulation 9. RAB37 has been identified to be a tumor suppressor and it regulated exocytosis of several proteins including TIMP metallopeptidase inhibitor 1 (TIMP1) 10, 11, secreted frizzled-related protein-1 (SFRP1) 12, thrombospondin-1 (TSP1) 13, and soluble ST2 14. In addition, promoter hypermethylation of* RAB37* was associated with metastasis 15. Thus, RAB37 had multiple functions in development, metabolism, immunity, and cancer.

As the core machinery of autophagy, the complex ATG5-ATG12-ATG16L1 was essential for LC3B lipidation and thus promoted autophagosome membrane biogenesis 6. Accordingly, upstream regulations of the complex ATG5-ATG12-ATG16L1 will be key steps in autophagy. Accumulated evidence showed that RAB37-mediated autophagy regulation was an important cellular event. Recently, our group found that RAB37 functioned as an organizer for autophagosome biogenesis 16, 17. RAB37 regulated autophagosome formation by modulating ATG5-ATG12-ATG16L1 complex assembly. GTP-bound RAB37 can interact directly with ATG5 and promoted interaction of the ATG5-ATG12 with ATG16L1. Multimeric RAB37-ATG5-ATG12-ATG16L1 complex was recruited and then lipidated LC3B to form active LC3B-PE, which accelerated autophagosome formation 16. However, transcription regulations of *RAB37* remain elusive. To further explore transcription activation of *RAB37*, in this study, we comparatively analyzed promoter activities of *RAB37* in humans, mice, and pigs, investigated transcription factors and their binding abilities to the promoters of *RAB37* genes in these species, screened polymorphism in pig *RAB37* gene, and identified *RAB37* alleles in pig populations worldwide. Further roles of these *RAB37* alleles in autophagy were analyzed. These results highlight the importance of transcription activation of *RAB37* in autophagy.

## Methods

### Antibodies and reagents

Anti-LC3B (Cat# 3868) was purchased from Cell Signalling Technology, Danvers, MA, USA. Anti-p62 (SQSTM1) (Cat# 18420-1-AP) was from Proteintech Group, Rosemont, IL, USA. Anti-FLAG antibody (Cat# F3165) was from Sigma-Aldrich, St Louis, MO, USA. Goat anti-rabbit IgG (H+L), horseradish peroxidase (HRP) conjugated antibody (Cat# 31460), goat anti-mouse IgG (H+L), HRP conjugated antibody (Cat# 31430), and TRITC-conjugated ImmunoPure goat anti-rabbit IgG (H+L) (A16071) were obtained from Invitrogen, Carlsbad, USA. DAPI (Cat# C1002) was purchased from Beyotime Institute of Biotechnology (Jiangsu, China). EBSS (Cat# SH30029.02) was from HyClone, Logan, USA.

### Plasmid constructs

Eight deletion fragments of the pig *RAB37* (ENSSSCT00025032574.1) promoter were amplified from pig genomic DNA, and cloned into pGL3-basic (E1751, Promega, Madison, WI, USA) vector digested by XhoΙ and HindⅢ. Full-length *EBF1* (ENSSSCT00000040359.2) and *EGR2* (ENSSSCT00025106300.1) of pig were cloned into pcDNA3.0 using HindⅢ/XhoΙ and HindⅢ/EcoRΙ double digestion to generate pCMV-pig-EBF1 and pCMV-pig-EGR2. Site-directed mutagenesis for the EBF1 and EGR2 binding sites were performed using the primers described in Table S1. PS-EBF1-MUT was used as a template for constructing PS-EBF1+EGR2-MUT.

Four different promoter fragments of pig *RAB37* containing relevant alleles were cloned into pGL3-basic vector (digested by XhoΙ and HindⅢ) to generate pGL3-*RAB37* (pGL3-*RAB37-a*, pGL3-*RAB37-b*, pGL3-*RAB37-c*, and pGL3-*RAB37-d*). These promoter fragments were cloned into RAB37-FLAG and RAB37-GFP 16, and replaced the transcription elements of the vector to construct RAB37-FLAG (RAB37-a-FLAG, RAB37-b-FLAG, RAB37-c-FLAG, and RAB37-d-FLAG) and RAB37-EGFP (RAB37-a-EGFP, RAB37-b-EGFP, RAB37-c-EGFP, and RAB37-d-EGFP). The primers and PCR conditions were listed in Table S1. All constructs were sequenced.

### In silico sequence and SNP analysis

Genomatix database (www.genomatix.de) was used to predict transcription factors in *RAB37* promoters. MethPrimer software (www.urogene.org) was used to predict the CpG islands in promoter with island size > 100, observed/expected ratios > 0.6, and GC percent > 50.0. JASPAR database (jaspar.genereg.net/) was used to search sequences and logos of transcription factors.

Genomic DNA samples from Meishan, Xihuzhou large white and Tongcheng pigs were used to amplify the *RAB37* promoter regions by polymerase chain reaction and primers described in Table S1. The sequences were aligned with available genomic data in *Sus scrofa* 11.1 using Ensembl and corresponding SNP position of the sequences was determined in the region of chr12: 6493414-6494172. Positions on the chromosome of SNP variations were at nt 6493746 (SNP1), nt 6493872 (SNP2), and nt 6493991 (SNP3). SNP sites were blasted against available SNP data in the Ensembl databases. SNP frequencies were calculated based on these data.

### Cell culture, transfection, and dual-luciferase reporter assays

COS-7, 293T and CHO cells were obtained from China Center for Type Culture Collection. COS-7 and 293T cells were cultured in DMEM (Cat# SH30022.01B, HyClone, Logan, USA) with 10% FBS (Cat# P30-330250, PAN-Biotech, Aidenbach, Germany). CHO cells were cultured in MEM (Cat# SH30023.01, HyClone, Logan, USA) with 10% FBS.

Dual-luciferase reporter assays were performed according to our previous protocols 18. Cells per 48-well were transfected with 0.5 μg recombinant construct and 10 ng pRL-TK (Cat# E2241, Promega) using LipofectamineTM 2000 (Cat# 11668027, Invitrogen). Luciferase activities were tested by a dual-luciferase reporter assay system (Promega, Madison, USA) and recorded by a Modulus Single Tube Multimode Reader (Turner Biosystems, Sunnyvale, USA) according to the manufacturer's protocol. The experiments were repeated at least 3 times, and analyzed as the means ± SD.

### Western blot analysis

Western blots were performed following our previous protocols 19. 293T cells were transfected with RAB37-FLAG or vector control. At 48 h after transfection, the whole protein extracts were extracted from the cells and used for SDS-PAGE. The gels were transferred onto a 0.45 μm PVDF membrane (Cat# NK0414, Roche Diagnostics, Indianapolis, IN, USA). The membranes were blocked with 5% skim milk powder (Cat# 1172GR100, BioFroxx Gmbh) in TBST and incubated with the primary antibody overnight at 4 °C followed by the horseradish peroxidase-conjugated secondary antibody at room temperature for at least 1 h. ECL Plus detecting reagents (Millipore, Billerica, MA, USA) were used to visualize the protein bands. ImageJ (version J2, NIH, Maryland, US) was used to analyze band intensity values.

### Immunofluorescence analysis

Immunofluorescence analysis was performed according to our previous study 16. CHO cells were cultured on glass coverslips. At 48 h after transfection using LipofectamineTM 2000, the cells were starved in EBSS medium for 1 h. After fixed and permeabilized, the samples were incubated with primary antibody overnight at 4 °C and subjected to indirect immunofluorescence secondary antibody. The DAPI was used to stain the nuclei. Confocal fluorescence microscope (Olympus, FV1000, Tokyo, Japan) was used to capture images.

### Statistics analysis

The data were presented as the means ± standard deviation from at least three independent experiments. One-way analysis of variance was tested for comparisons among more than two groups. Statistics analysis was performed using GraphPad Prism 7 software package (GraphPad Software, La Jolla, USA). For all analyses, *P* value < 0.05 was considered to be statistically significant.

## Results

### EBF1 and EGR2 activate the pig *RAB37* promoter

**Figure 1:**
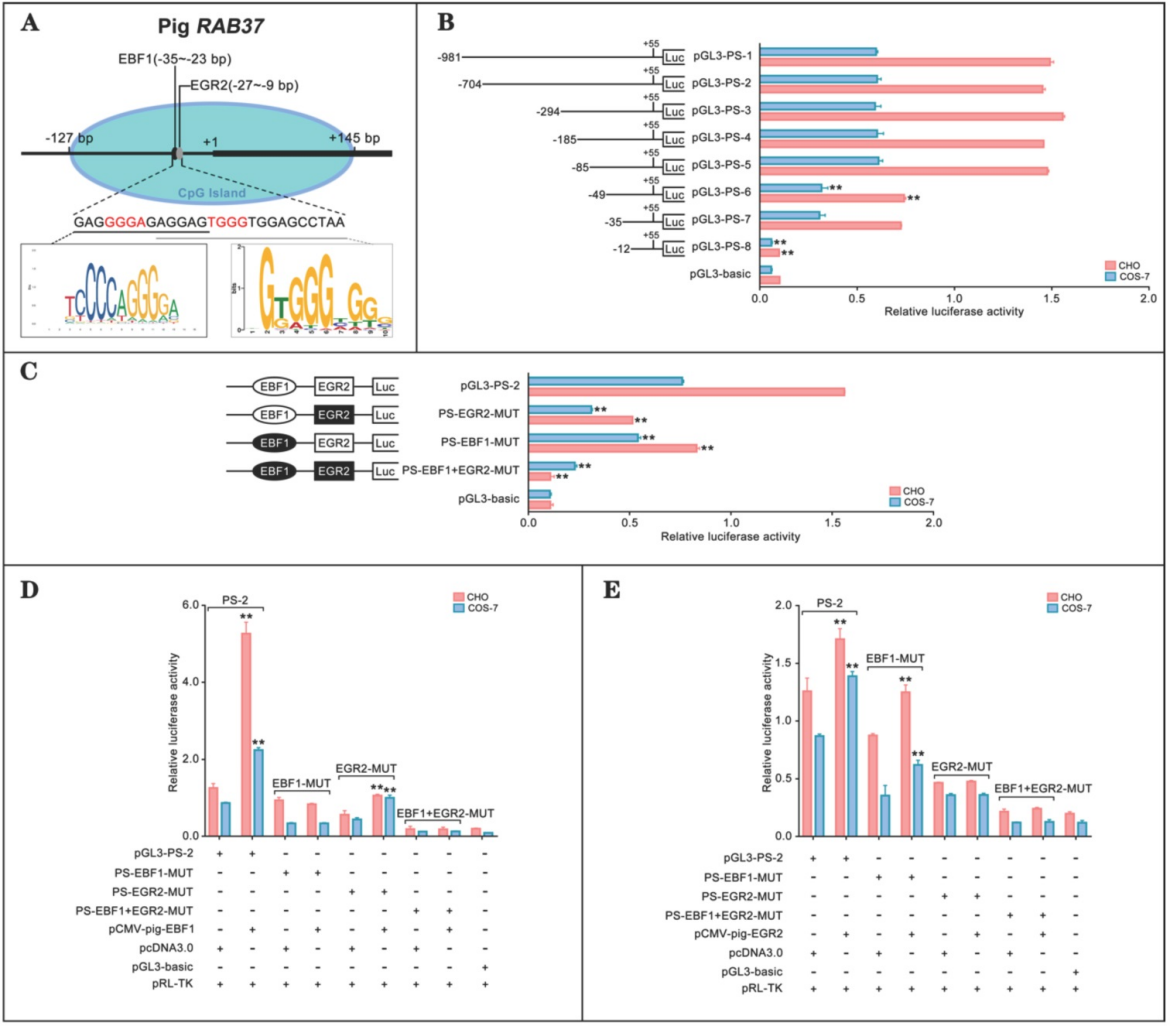
** Promoter activity and transcription activation of pig *RAB37*.** (**A**) Schematic diagram of the pig *RAB37* promoter. An EGR2 binding site and an EBF1 binding site are located in the CpG island (blue oval). Sequence logos of EBF1 and EGR2 binding sites are shown in the lower panel, and logos are based on JASPAR database. (**B**) Luciferase assays showing activities of a series of deleted constructs in both CHO and COS-7 cells. Left panel indicates each deleted mutant linked with the luciferase gene in the pGL3-basic vector. Right panel shows relative activities of these deleted constructs, as determined by luciferase assays. One-way ANOVA was performed. **P < 0.01. (**C**) Luciferase assays of point mutations in core promoter. The pGL3-PS-2 construct of 704 bp was used as a basic construct for the analysis. Luciferase assays were used to determine the relative activities. The intact binding sites of EBF1 and EGR2 are indicated by open ovals and boxes respectively. The filled ovals and boxes show the corresponding mutations. The pGL3-basic vector was used as a control. One-way ANOVA was performed. **P < 0.01. (**D**) Overexpression of EBF1 activates the pig *RAB37* promoter. In total, 0.4 mg pGL3-PS-2 or its site mutants (PS-EBF1-MUT, PS-EGR2-MUT, or PS-EBF1+EGR2-MUT) were cotransfected with 0.1 mg *EBF1* expressing plasmid (pCMV-pig-EBF1). EBF1 overexpression can increase the promoter activity, except PS-EBF1-MUT and PS-EBF1+EGR2-MUT. One-way ANOVA was performed. **P < 0.01. (**E**) Overexpression of EGR2 activates the pig *RAB37* promoter. In each transfection, 0.4 mg pGL3-PS-2 or its site mutants (PS-EBF1-MUT, PS-EGR2-MUT, or PS-EBF1+EGR2-MUT) were cotransfected with 0.1mg *EGR2* expression plasmid (pCMV-pig-EGR2). EGR2 overexpression increases the promoter activity, except the PS-EGR2-MUT and PS-EBF1+EGR2-MUT. One-way ANOVA was performed. **P < 0.01.

To explore transcription regulation of *RAB37*, we analyzed potential transcription factors to activate* RAB37* transcription in pig. CpG island and transcription factors were first predicted. An EBF1 binding site (-35 bp to -23 bp) and an E2F1 binding site (-27 bp to -9 bp) were detected in a CpG island in the potential promoter in pig (Figure 1A). Deletion analysis and luciferase analysis showed that sequence from -85 bp to -12 bp in the 5' flanking region was essential for the promoter activity (Fig. 1B). To determine roles of these two sites for transcription factors EBF1 and EGR2, site-directed mutants were constructed using the wild-type pGL3-PS-2 as a template. Compared with the pGL3-PS-2 construct, activities of single mutants PS-EBF1-MUT and PS-EGR2-MUT, and double mutant PS-EBF1+EGR2-MUT were significantly decreased, while the activities were obviously low in double mutant PS-EBF1+EGR2-MUT (Figure 1C). These results suggested that the binding sites for the transcription factors EBF1 and EGR2 were important for the promoter activity of pig *RAB37*.

The binding abilities of EBF1 and EGR2 to the corresponding sites were further tested by luciferase assays, which showed that EBF1 could increase the luciferase activities of wild-type pGL3-PS-2 and mutant PS-EGR2-MUT, but not its single mutant PS-EBF1-MUT and double mutant PS-EBF1+EGR2-MUT (Figure 1D). On the other hand, EGR2 could also activate the activities of wild-type pGL3-PS-2 and single mutant PS-EBF1-MUT, but had no contribution to the activities of single mutant PS-EGR2-MUT and double mutant PS-EBF1+EGR2-MUT (Figure 1E). Thus, EBF1 and EGR2 were the transcription factors of the pig *RAB37* promoter.

### Transcription factor-associated evolution in *RAB37* promoter

**Figure 2:**
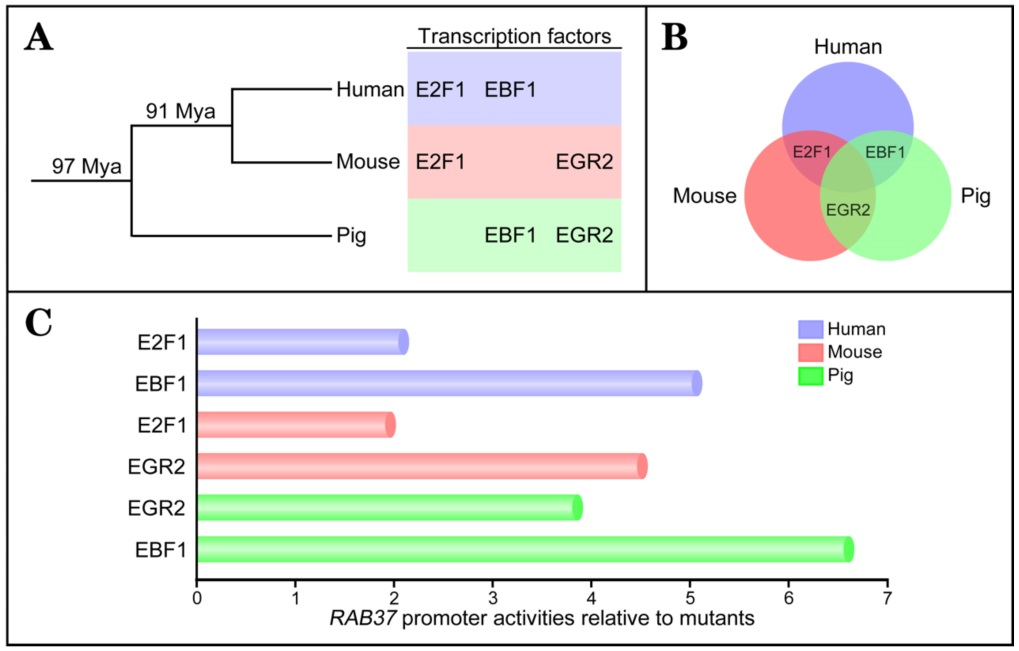
** Evolution of transcription factors in *RAB37* promoter.** (**A**) Phylogenetic relationship of transcription factors EGR2, E2F1, and EBF1 in the promoter of *RAB37* gene. Mya, million years ago. (**B**) Venn diagram showing conservation of transcription factors EGR2, E2F1, and EBF1 for *RAB37* among human (blue circle), mouse (red circle), and pig (green circle). E2F1 is a common transcription factor between human and mouse, EBF1 is a common transcription factor between human and pig, EGR2 is a common transcription factor between mouse and pig. (**C**) Comparisons of transcriptional activation abilities of transcription factors EGR2, E2F1, and EBF1 in the* RAB37* promoter among human (blue), mouse (red) and pig (green). The* RAB37* promoter activities were determined by luciferase assays in COS-7 cells and calculated by relative values to their corresponding point mutants.

To investigate conservation of transcription factors in regulation of *RAB37* gene, the same approaches were used to identify transcription factors to activate the *RAB37* promoters in human and mouse. The analysis results showed that binding sites for the transcription factors EBF1 and E2F1 were important for the promoter activity of human *RAB37* (Supplementary Figure S1), while EGR2 and E2F1 were the transcription factors of the mouse *Rab37* promoter (Supplementary Figure S2). To realize phylogenetic relationship of *RAB37* genes in human, mouse, and pig, phylogenetic analysis of binding sites of the transcription factors in the promoters were performed among these species. Based on phylogeny time of mammals 20, the evolution analysis showed that EBF1 and EGR2 were evolved in pig ~97 Mya, and EBF1 was replaced by E2F1 in mouse ~91 Mya. However, EGR2 site was degenerated and both EBF1 and E2F1 sites were evolved in human (Figure 2A). Venn diagram showed alternative usage of two transcription factors from EGR2, E2F1, and EBF1 in *RAB37* promoters among human, mouse, and pig (Figure 2B).

Comparisons of transcriptional activation abilities of transcription factors EGR2, E2F1, and EBF1 in the *RAB37* promoters among human, mouse, and pig revealed that each species had a dominant transcription factor (EBF1 in human and pig; EGR2 in mouse), while another transcription factor was also essential for transcription activation of the *RAB37* gene (Figure 2C). These results suggested earlier evolved transcription factors EBF1 and EGR2 had a dominant contribution to *RAB37* transcription, although divergent evolution of binding sites of transcription factors in the *RAB37* promoters existed in different lineages.

### Artificial selection generates *RAB37* alleles in pig populations

**Figure 3:**
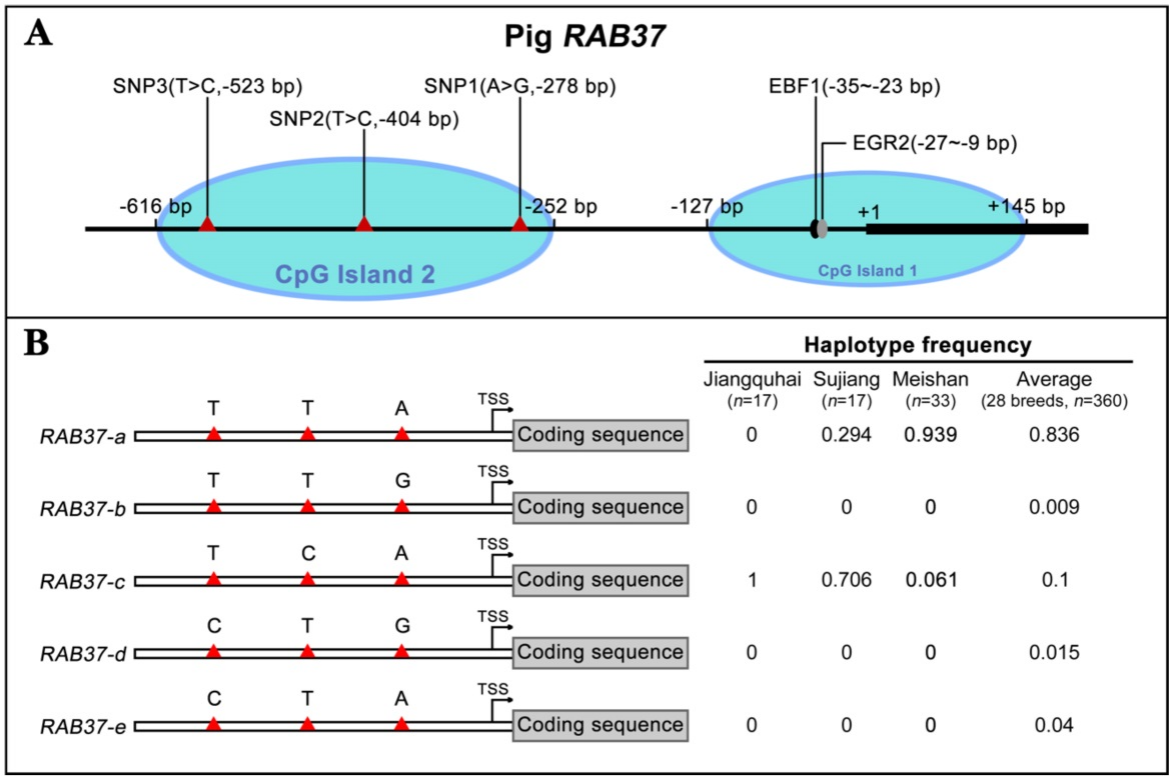
** Identification of *RAB37* SNPs and haplotypes in pig population.** (**A**) Schematic diagram of the SNPs in the pig *RAB37* promoter. Three SNPs (A>G, -278 bp; T>C, -404 bp; T>C, -523 bp) are in CpG island 2, whereas EGR2 and EBF1 binding sites are located in CpG island 1. The blue ovals indicate the CpG islands. (**B**) Identification of *RAB37* haplotypes in pig population. In total, 5 haplotypes of* RAB37* were detected from 28 pig breeds. Haplotypes frequencies were calculated based on all available data of 28 pig breeds.* RAB37-a* is a major haplotype, *RAB37-b* has a variation A>G in SNP1 site, *RAB37-c* has T>C in SNP2 site, *RAB37-e* has T>C in SNP1 site, while *RAB37-d* has A>G in SNP1 site as well as T>C in SNP3 site. Haplotype frequencies in Jiangquhai, Sujiang, Meishan pigs and average of all 28 breeds are shown in the right panel.

To further explore polymorphism in pig *RAB37*, genomic sequences of the pig *RAB37* promoter were PCR amplified from genomic DNA samples of Meishan, Tongcheng, and Xihuzhou large white pigs. Sequencing analysis revealed 3 SNP sites in the CpG island 2 (Figure 3A). SNP1 (A>G) was located at 278 bp upstream of transcriptional start site (TSS), SNP2 (T>C) ws at 404 bp upstream of the TSS, SNP3 (T>C) at 523 bp upstream of the TSS (Figure 3A). Frequency of these SNPs in 28 different breeds, including both Asian and European wild pigs, 17 Chinese breeds, and 4 breeds with large white descent, and 5 Western breeds, were further analyzed (Table 1), which showed that biased frequency distributions of these SNPs in these breeds.

Haplotype analysis of these SNPs showed that 5 haplotypes existed in these pig breeds (Figure 3B). *RAB37-a* was a major haplotype. Compared with *RAB37-a*,* RAB37-b* had a variation A>G in SNP1 site, *RAB37-c* with T>C in SNP2 site, and *RAB37-e* with T>C in SNP3 site, while* RAB37-d* had A>G in SNP1 site as well T>C in SNP3 site. In pig populations, these haplotypes represented 5 alleles of* RAB37* gene.

**Figure 4:**
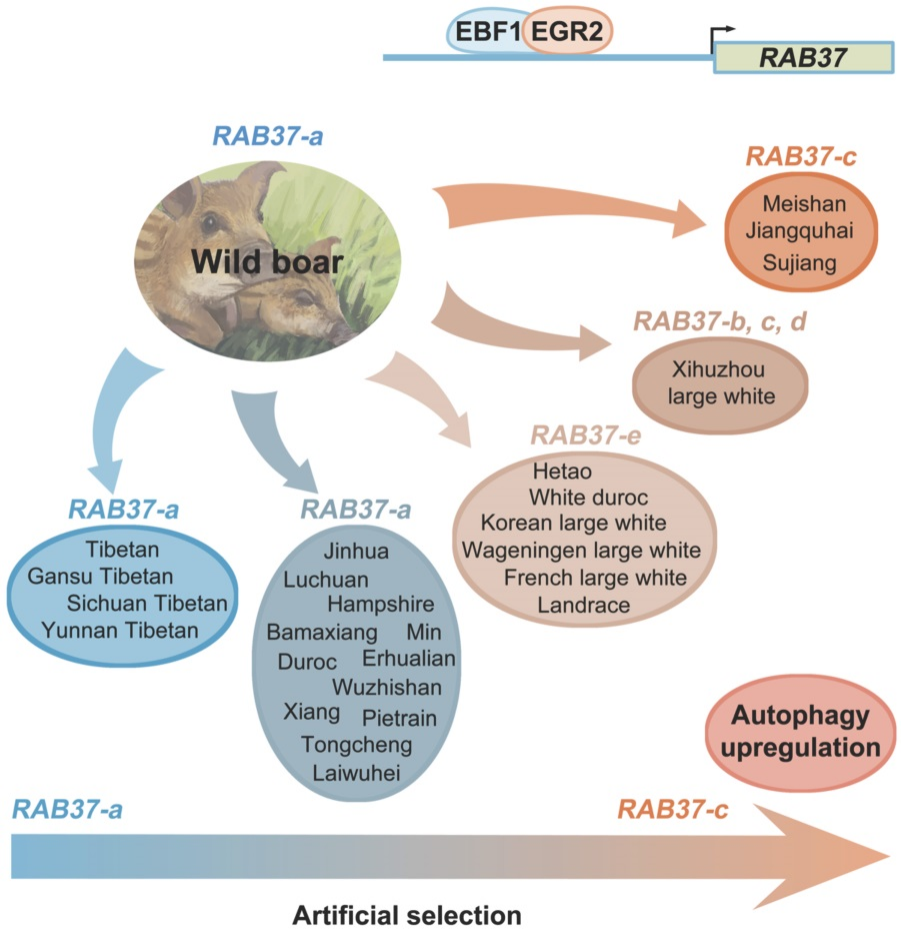
** Artificial selection generates *RAB37* multiple alleles in pig population.** Wild boars have only wild-type *RAB37-a*. Tibetan pigs experienced a weak pressure of artificial selection and retain only *RAB37-a*. In domestic pigs, artificial selection generates new alleles *RAB37-b*, *RAB37-c, RAB37-d,* and* RAB37-e* in some breeds*,* while* RAB37-a* is still only allele in many breeds. In addition, transcription factors EBF1 and EGR2 have be selected to activate *RAB37* transcription in the pig lineage.

SNP haplotyping revealed both Asian and European wild pigs, Tibetan pigs, most of local breeds in China, and Duroc, and Hampshire and Pietrain pigs of Western breeds, had only *RAB37-a*, indicating that *RAB37-a* was a wild-type allele. Landrace, White duroc, Hetao pigs, and French-, Korean-, and Wageningen-large white pigs had a small proportion of* RAB37-e*, in addition to wild-type* RAB37-a*. Xihuzhou large white pigs from China had *RAB37-b, RAB37-c, RAB37-d,* and the wild-type* RAB37-a*, suggesting a mixed population. Interestingly, Chinese local breeds, Jiangquhai, Sujiang, and Meishan pigs had *RAB37-c,* in addition to wild-type* RAB37-a,* and Jiangquhai pigs had only *RAB37-c* in particular (Table 1; Figure 3B). Thus, wild boars from both Chinese and European, and Tibetan pigs had only wild-type *RAB37-a*, new alleles *RAB37-b*,-*e* were mainly detected in domestic pig breeds, with 100% allele *RAB37-c* in Jiangquhai pigs (Figure 4). These data suggested that *RAB37* multiple alleles were generated by artificial selection in pig populations.

### Promoter activities and roles of *RAB37* alleles in autophagy

**Figure 5:**
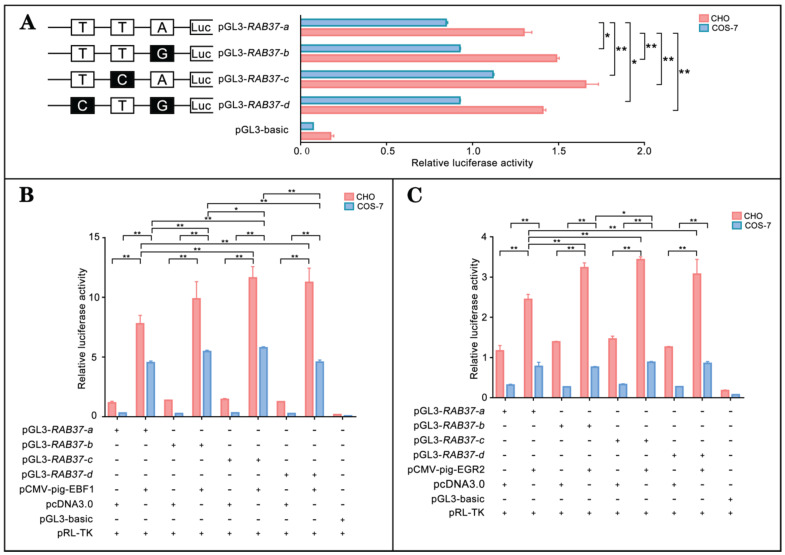
** Promoter activities and transcription activation of *RAB37* alleles in pig.** (**A**) Luciferase assays showing promoter activities of *RAB37* alleles in both CHO and COS-7 cells. SNPs are indicated by boxes, while the filled boxes show corresponding SNPs with lower frequency relative to those in allele* RAB37-a*. Right panel shows relative activities of these promoters containing SNPs, as determined by luciferase assays. One-way ANOVA was performed. *P < 0.05; **P < 0.01. (**B**) Overexpression of EBF1 activates these allele promoters. In total, 0.4 mg pGL3-*RAB37-a* or its SNP mutants (pGL3-*RAB37-b*, pGL3-*RAB37-c*, or pGL3-*RAB37-d*) were cotransfected with 0.1 mg *EBF1* expressing plasmid (pCMV-pig-EBF1). EBF1 overexpression can increase the activities of all these promoters containing SNPs with the highest in pGL3-*RAB37-c*. One-way ANOVA was performed. *P < 0.05; **P < 0.01. (**C**) Overexpression of EGR2 activates these allele promoters. In total, 0.4 mg pGL3-*RAB37-a* or its site mutants (pGL3-*RAB37-b*, pGL3-*RAB37-c*, or pGL3-*RAB37-d*) were cotransfected with 0.1 mg *EGR2* expression plasmid (pCMV-pig-EGR2). EGR2 overexpression increases activities of these allele promoters containing SNPs with the highest in pGL3-*RAB37-c*. One-way ANOVA was performed. *P < 0.05; **P < 0.01.

To realize roles of these alleles, promoter regions of the alleles were linked with luciferase gene in the pGL3-basic vector and their activities were tested. The analysis showed that luciferase activities of the constructs with SNP mutants (pGL3-*RAB37-b*, pGL3*-RAB37-c*, pGL3-*RAB37-d*) were higher than that of wild-type pGL3-*RAB37-a* in CHO cells (Figure 5A). To further explore influence of SNP mutants on transcription activation of the transcription factors, these constructs were cotransfected with EBF1 or EGR2 in CHO and COS-7 cells, and luciferase activities were tested. The luciferase reporter analysis showed that both EBF1 and EGR2 could increase promoter activities of all these constructions with the highest in pGL3-*RAB37-c* (Figure 5B and 5C). These data suggested that these SNP variations in the promoter could upregulate activity of the pig *RAB37* promoter.

**Figure 6:**
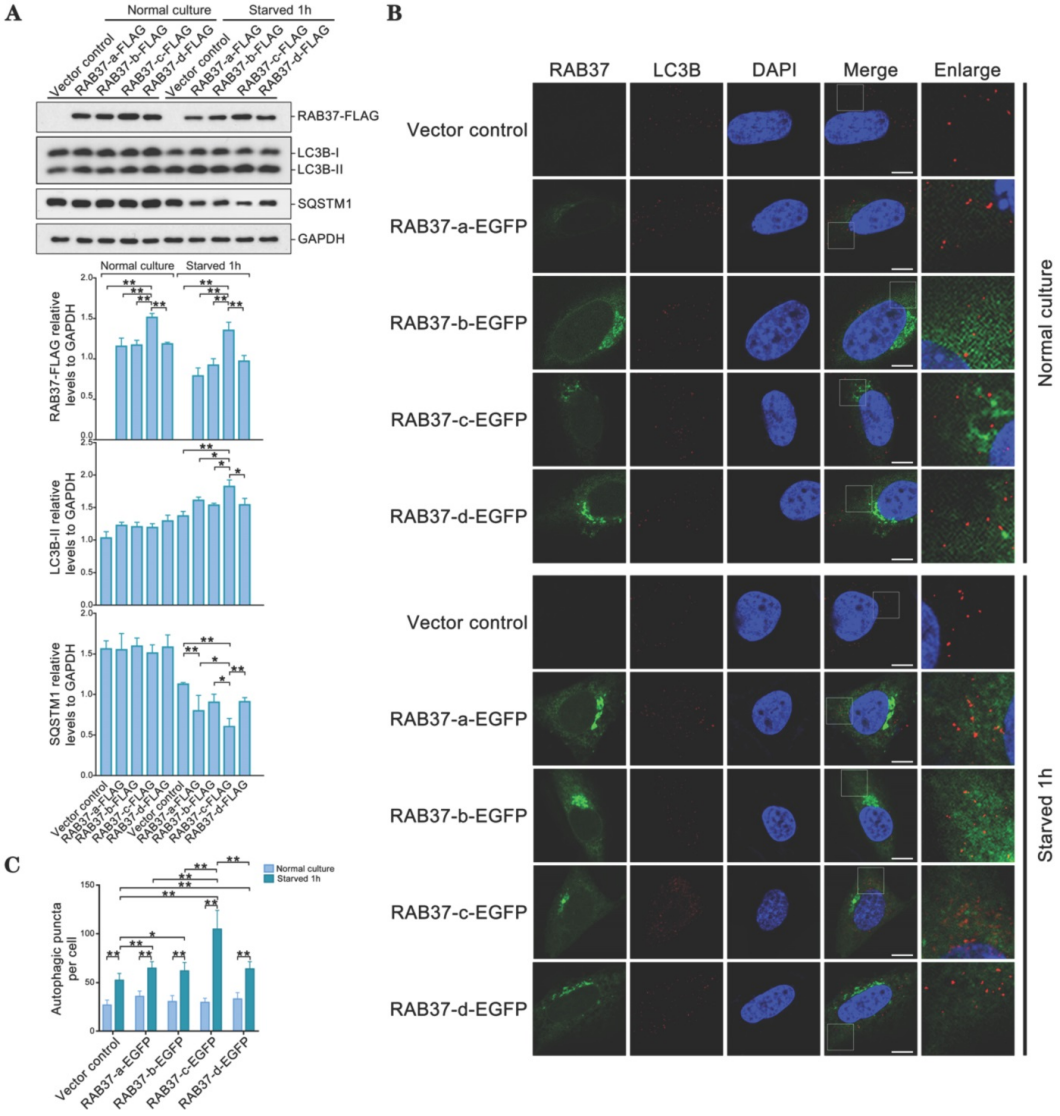
** Alleles of pig *RAB37* promote autophagosome formation.** (**A**) Western blot analysis of RAB37-FLAG, LC3B, and SQSTM1 in 293T cells. The cells were transfected with RAB37-FLAG constructs (RAB37-a-FLAG, RAB37-b-FLAG, RAB37-c-FLAG, RAB37-d-FLAG, and negative control vector) and cultured in normal (control) or EBSS medium for 1 h, respectively. The cell lysates were analyzed by immunoblotting with antibodies as indicated. GAPDH was used as an endogenous control. Bottom panels: western blots quantified for RAB37-FLAG/GAPDH ratio, LC3BII/GAPDH ratio and the SQSTM1/GAPDH ratio. Data are presented as means ± S.D. *P < 0.05, **P < 0.01 (n = 3 independent experiments). (**B**) Immunofluorescence analysis of RAB37-EGFP and LC3B in CHO cells. Representative images of the cells transfected with these RAB37-EGFP constructs (RAB37-a-EGFP, RAB37-b-EGFP, RAB37-c-EGFP, and RAB37-d-EGFP). The cells were cultured in normal (control) or EBSS medium for 1 h, respectively. Endogenous LC3B (red) was detected first by anti-LC3B and then TRITC-conjugated ImmunoPure goat anti-rabbit IgG (H + L). The nuclei were stained by DAPI (blue). Vector control is a negative control without promoter. Single channel (red, green or blue) and merged images were taken by confocal microscopy. The enlarged images originated from the white squares in merge panels. Scale bar, 10 µm. (**C**) Statistics of autophagic puncta per cell from A. The dots were counted from 20 cells. *P < 0.05; **P < 0.01.

As RAB37 can promote autophagy 16, we further compared roles of these alleles in enhancing autophagy. *RAB37* alleles with various SNP variations were transfected into 293T cells and starved for 1 h. Western blots showed that LC3B-II was upregulated, while downstream substrate SQSTM1 was downregulated upon starvation (Figure 6A). *RAB37* alleles with various SNPs were overexpressed in CHO cells and endogenous LC3B puncta were further analyzed under starvation. In comparison with normal culture, autophagic puncta in *RAB37-c* were significantly higher than those in *RAB37-a, RAB37-b,* and *RAB37-d*, while these alleles could enhance autophagy upon starvation (Figure 6B and 6C). These results suggested these SNP variations in RAB37 promoter promoted autophagy.

## Discussion

Considering the importance of RAB37 as an organizer for autophagosome biogenesis, mechanistic insights into transcription regulation of *RAB37* will provide better understanding of roles and mechanisms of autophagy in cellular homeostasis and development. In this study, we characterized* RAB37* promoters in humans, mice, and pigs. Transcription factors and their binding abilities to the promoters were determined. We further screened SNPs in pig *RAB37* gene and identified *RAB37* alleles in pig populations from both China and Europe. In addition, roles of *RAB37* alleles in autophagy were confirmed. These findings provide better understanding of transcription activation of *RAB37* and suggest *RAB37* as a potential gene for artificial selection of autophagy regulation in pig breeding practice.

By comparative analysis of promoter activities of *RAB37* genes among humans, mice and pigs, a set of related transcription factors were identified. Transcription factors EBF1 and E2F1 were essential for transcription activation of *RAB37* gene in human, while EBF1 was a dominant regulator in* RAB37* in both human and pig. EBF1 belongs to a transcription factors family containing an atypical zinc finger DNA binding domain and a nonbasic helix-loop-helix dimerization domain, which mainly regulated adipocyte morphology and lipolysis 21 and B cell differentiation 22. EBF1 can interact with the methylcytosine dioxygenase enzyme TET2 in several cancer types, suggesting a role of EBF1 in regulating DNA methylation 23. Indeed, EBF1 binding site in *RAB37* promoter is located in a CpG island in both human and pig, hinting a role of epigenetic regulation of EBF1 in *RAB37* transcription. Considering a key role of RAB37 in regulation of autophagosome formation via modulating ATG5-ATG12-ATG16L1 complex assembly 16, 17, EBF1-mediated RAB37 pathway is probably involved in cellular homeostasis. EBF1 as a transcription factor of *RAB37* suggests their synergistic role in autophagy regulation. EBF1 knockdown affected lipid and glucose levels, thus energy metabolism 21, which supports importance of EBF1-mediated RAB37 pathway in autophagy regulation and cellular homeostasis.

In addition to EBF1, co-regulation of transcription factors EGR2 and E2F1 differentially evolved for *RAB37* genes in different lineages of human, mouse, and pig. Further, a dominant transcription factor (EBF1 in human and pig; EGR2 in mouse) for *RAB37* genes was acquired in these species. These data suggest that these transcription factors regulate RAB37 via transcription factor-associated evolution in RAB37 promoter for maintaining cellular homeostasis. These transcription factors can also directly regulate transcription of autophagy genes. For example, E2F1 can bind to the promoter regions of autophagy genes LC3B, ATG1, and DRAM to activate transcription of these genes, thus enhanced autophagy 24. In human adipocytes, EBF1 chromatin immunoprecipitation showed potential EBF1 target genes including autophagy related genes *ATG12, ATG16L2, ATG9B, ULK3*, *SQSTM1* and *RAB37* 21. Thus, transcription factors EBF1 and E2F1 maintain cellular homeostasis by either directly regulating some autophagy genes or indirectly modulating autophagy via activating *RAB37*. These two modes of regulation probably exert roles synergistically, which is important for maintaining cellular homeostasis upon different forms of stress. Further studies are needed to clarify this mechanism.

Long-term artificial selection of female fecundity and meat production has changed gene expression pattern in pig breeding 25. Here we further showed that the artificial selection generated SNP variations in the pig *RAB37* promoter, in addition to transcription factors EBF1 and EGR2 selected to activate *RAB37* promoter in the pig lineage. Three SNPs are located in second CpG island upstream of core promoter of *RAB37*, which could affect binding of EBF1 and EGR2 to *RAB37* promoter by mediating spatial structure of transcription complex. These SNP variations resulted in at least 5 haplotypes, representing 5 alleles of* RAB37*. Of them, *RAB37-c* acquired the highest ability to activate expression in comparison with the other promoters, thus enhanced autophagy efficiently. A large scale of population genome analysis from both China and Europe demonstrated that artificial selection led to *RAB37* multiple alleles in pig population (Figure 4). Both Chinese and European wild boars have only wild-type *RAB37-a*. Tibetan pigs also have only *RAB37-a*, as experienced a weak pressure of artificial selection in the Tibetan Plateau. However, new alleles *RAB37-b*, *RAB37-c, RAB37-d,* and* RAB37-e* appeared in domestic pig breeds. In particular, Jiangquhai pig, a Chinese local breed with a high litter size (n=13.5 at third birth), acquired 100% frequency of the allele *RAB37-c*. Considering a high female fecundity in Jiangquhai pigs and Meishan pigs, the allele *RAB37-c* could be artificially selected for autophagy regulation. Further analysis to illuminate the molecular mechanism will help in genetics and breeding practice in pigs.

## Conclusions

In this study, we performed a comparative analysis of promoter activities and related transcription factors of *RAB37* in humans, mice and pigs. Transcription factors and their binding abilities to the promoters were determined. SNPs in the pig *RAB37* gene were screened and multiple alleles of* RAB37* in pig populations from both China and Europe were identified. Population genomics showed a role of artificial selection in variations of these alleles. Furthermore, roles of *RAB37* alleles in autophagy regulation were confirmed. These findings provided a better understanding of transcription activation of *RAB37* and suggested *RAB37* as a potential gene for artificial selection of autophagy regulation in pig breeding practice.

## Supplementary Material

Supplementary figures and tables.

## Figures and Tables

**Table 1:** Haplotype frequency of* RAB37* in pig breeds

Breed	*n*	*RAB37-a* (TTA)	*RAB37-b* (TTG)	*RAB37-c* (TCA)	*RAB37-d* (CTG)	*RAB37-e* (CTA)
**Wild boar**						
Asian wild boar	10	1	0	0	0	0
European wild boar	17	1	0	0	0	0
**Local breed in China**						
Tibetan	12	1	0	0	0	0
Gansu Tibetan	10	1	0	0	0	0
Sichuan Tibetan	12	1	0	0	0	0
Yunnan Tibetan	12	1	0	0	0	0
Bamaxiang	6	1	0	0	0	0
Jinhua	6	1	0	0	0	0
Luchuan	6	1	0	0	0	0
Laiwuhei	6	1	0	0	0	0
Min	6	1	0	0	0	0
Wuzhishan	6	1	0	0	0	0
Xiang	2	1	0	0	0	0
Tongcheng	18	1	0	0	0	0
Hetao	6	0.917	0	0	0	0.083
Erhualian	21	1	0	0	0	0
Meishan	33	0.939	0	0.061	0	0
Sujiang	17	0.294	0	0.706	0	0
Jiangquhai	17	0	0	1	0	0
**Large white descent**						
Xihuzhou large white	26	0.481	0.115	0.192	0.212	0
French large white	36	0.931	0	0	0	0.069
Korean large white	14	0.786	0	0	0	0.214
Wageningen large white	14	0.5	0	0	0	0.5
**Europe-breed**						
Duroc	22	1	0	0	0	0
White duroc	2	0.75	0	0	0	0.25
Hampshire	2	1	0	0	0	0
Pietrain	5	1	0	0	0	0
Landrace	16	0.938	0	0	0	0.062
**Average**		**0.836**	**0.009**	**0.1**	**0.015**	**0.04**
