# Supplementary material for: RAB37 multiple alleles, transcription activation and evolution in mammals
Source: Int J Biol Sci. 2020 Sep 19;16(15):2964–73. doi: 10.7150/ijbs.47959 (PMC7545722; doi:10.7150/ijbs.47959)
Supplement: Supplementary file 1 — Supplementary figures and tables. [file ijbsv16p2964s1.pdf]

Supplementary information

Methods for plasmid constructs

Eight deletion fragments of the human *RAB37* (NM_001006638.3) promoter were amplified from human genomic DNA, and cloned into the pGL3-basic vector double-digested by XhoI and HindIII. Full-length *EBF1* (NM_001324101.2) and *E2F1* (NM_005225.3) of human were cloned into pcDNA3.0 using HindIII/XhoI and HindIII/EcoRI to generate pCMV-human-EBF1 and pCMV-human-E2F1. Site-directed mutagenesis for the EBF1 (EBF1-1 and EBF1-2) and E2F1 binding sites were performed using the primers described in Table S1. HS-EBF1-1-MUT was used as a template for constructing HS-EBF1-1/2-MUT, and then the HS-EBF1-1/2-MUT was used as the template to construct HS-EBF1-1/2+E2F1-MUT.

Twelve deletion fragments of the mouse *Rab37* (NM_021411.4) promoter were amplified from mouse genomic DNA, and cloned into pGL3-basic vector double-digested by MluI and XhoI. Full-length *Egr2* (NM_010118.3) and *E2f1* (NM_007891.5) of mouse were cloned into pcDNA3.0 using EcoRI/XhoI and HindIII/EcoRI to generate pCMV-mouse-EGR2 and pCMV-mouse-E2F1. Site-directed mutagenesis for the EGR2 and E2F1 binding sites was performed using the primers described in Table S1. MS-EGR2-MUT was used as a template for constructing MS-EGR2+E2F1-MUT.

Figure S1

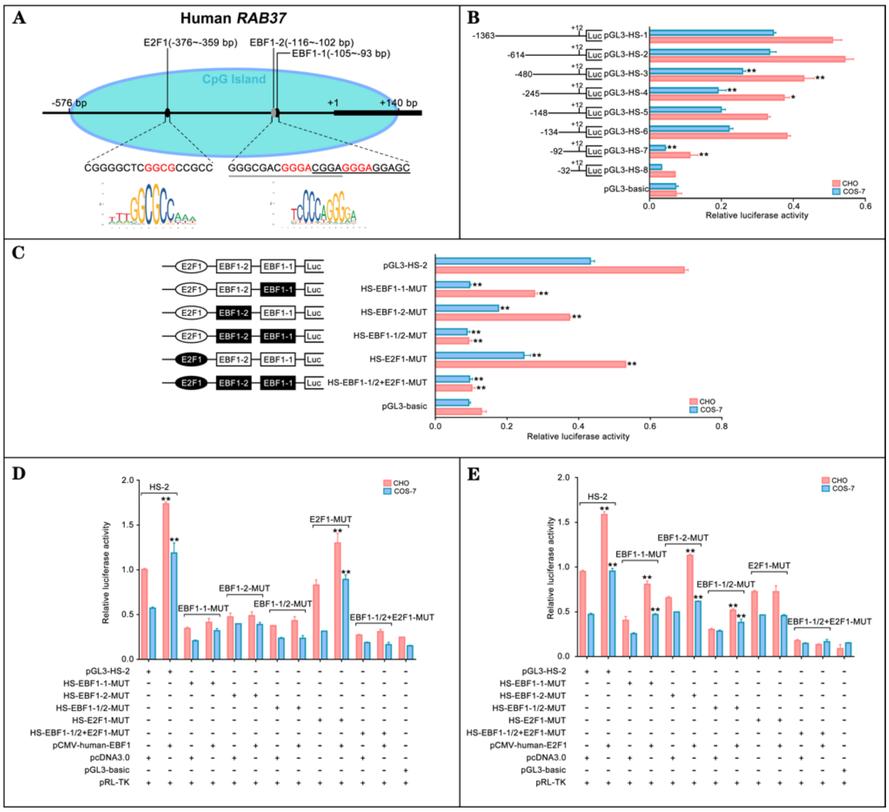

Figure S1. Promoter activity and transcription activation of human *RAB37*.

(A) Schematic diagram of the human *RAB37* promoter. An E2F1 binding site and two EBF1 binding sites are located in a CpG island (blue oval) in the promoter. Sequences and logos of EBF1 and E2F1 binding sites are shown in the lower panel, and logos are based on JASPAR

database.

(B) Luciferase assays of activities of a series of deleted constructs of the human *RAB37* promoter. Left panel indicates each deleted mutant linked with luciferase gene in the pGL3-basic vector. Right panel shows relative luciferase activities of these deleted constructs in both CHO and COS-7 cells, and pGL3-basic was used as a control. One-way ANOVA was performed. *, P < 0.05; **, P < 0.01.

(C) Point mutation analysis of the core promoter using luciferase assays. The pGL3-HS-2 construct of 614 bp was used as a basic construct for the analysis. Luciferase assays were used to determine the relative activities. The intact binding sites of E2F1, EBF1-1 and EBF1-2 are indicated by open ovals and boxes, respectively. The filled ovals and boxes show the corresponding mutations. The pGL3-basic vector was used as a control. One-way ANOVA was performed. **, P < 0.01.

(D) Overexpression of EBF1 activates the human *RAB37* promoter. In each transfection, 0.4 mg pGL3-HS-2 or its site mutants (HS-EBF1-1-MUT, HS-EBF1-2-MUT, HS-EBF1-1/2-MUT, HS-E2F1-MUT, or HS-EBF1-1/2+E2F1-MUT) were cotransfected with 0.1 mg *EBF1* expressing plasmid (pCMV-human-EBF1). EBF1 overexpression can only increases the activities of pGL3-HS-2 and HS-E2F1-MUT. One-way ANOVA was performed. **, P < 0.01.

(E) Overexpression of E2F1 activates the human *RAB37* promoter. In each transfection, 0.4 mg pGL3-HS-2 or its site mutants (HS-EBF1-1-MUT, HS-EBF1-2-MUT, HS-EBF1-1/2-MUT, HS-E2F1-MUT, or HS-EBF1-1/2+E2F1-MUT) were cotransfected with 0.1 mg *E2F1* expressing plasmid (pCMV-human-E2F1). E2F1 overexpression increases the promoter activity, except the mutants HS-E2F1-MUT and HS-EBF1-1/2+E2F1-MUT. One-way ANOVA was performed. **, P < 0.01.

Figure S2

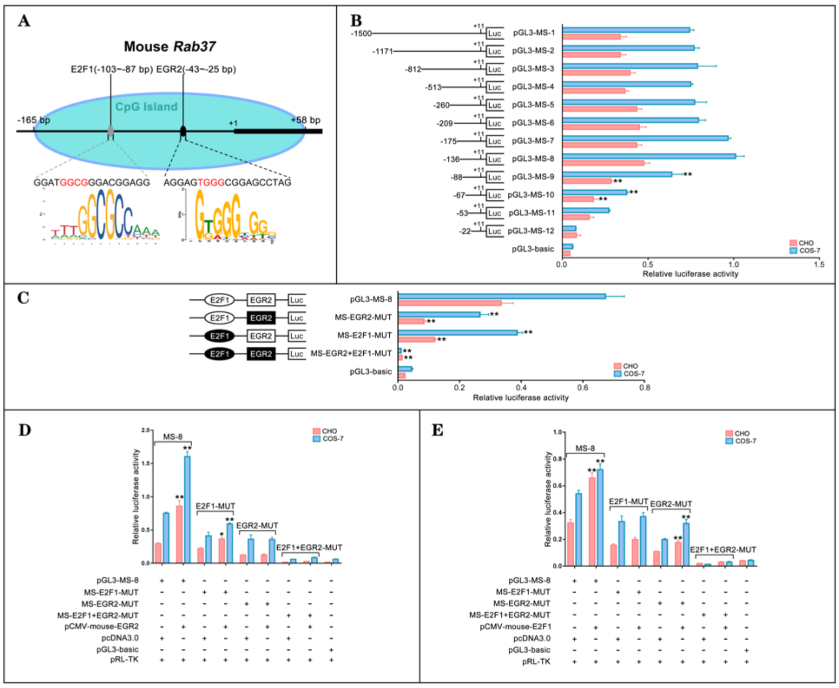

Figure S2. Promoter activity and transcription activation of mouse *Rab37*.

(A) Schematic diagram of the mouse *Rab37* promoter, in which an E2F1 binding site and an

EGR2 binding site are detected. The blue oval represents the CpG island. Sequence logos of E2F1 and EGR2 binding sites are shown in the lower panel, and logos are based on JASPAR database.

(B) Luciferase assays showing the activities of a series of deleted constructs in both CHO and COS-7 cells. Left panel indicates these deleted mutants. Right panel shows the relative activities of these constructs. One-way ANOVA was performed. **, $P < 0.01$.

(C) Luciferase assays of point mutations in core promoter. The pGL3-MS-8 of 136 bp was used as a basic construct. The intact binding sites of E2F1 and EGR2 are indicated by open ovals and boxes respectively. The filled boxes and circles show the corresponding mutations. The pGL3-basic vector was used as a control. One-way ANOVA was performed. **, $P < 0.01$.

(D) Overexpression of EGR2 activates the mouse *Rab37* promoter. In total, 0.4 mg pGL3-MS-8 or its site mutants (MS-EGR2-MUT, MS-E2F1-MUT, or MS-EGR2+E2F1-MUT) were cotransfected with 0.1 mg *Egr2* expressing plasmid (pCMV-mouse-EGR2). EGR2 overexpression increases the promoter activity, except MS-EGR2-MUT and MS-EGR2+E2F1-MUT. One-way ANOVA was performed. *, $P < 0.05$; **, $P < 0.01$.

(E) Overexpression of E2F1 activates the mouse *Rab37* promoter. In each transfection, 0.4 mg pGL3-MS-8 or its site mutants (MS-EGR2-MUT, MS-E2F1-MUT, or MS-EGR2+E2F1-MUT) were cotransfected with 0.1 mg *E2f1* expressing plasmid (pCMV-mouse-E2F1). E2F1 overexpression can increase the promoter activity, except the MS-E2F1-MUT and MS-EGR2+E2F1-MUT. One-way ANOVA was performed. **, $P < 0.01$.

Table S1. Primer sequences and PCR conditions

Genes/fragments	GenBank access No.	Primer sequence (5'-3')	Tm (°C)
**Human**			
RAB37(pGL3-HS-1)	NM_001006638.3	F: AATCTCGAGCCTCACCTCCACACCTA<br>R: AATAAGCTTTGGACGAGAGGTGAGCA	57
RAB37(pGL3-HS-2)	NM_001006638.3	F: AATCTCGAGGGGGAAATGAGAGGTGA<br>R: AATAAGCTTTGGACGAGAGGTGAGCA	57
RAB37(pGL3-HS-3)	NM_001006638.3	F: AATCTCGAGGCCGGGTGACTTAACAGA<br>R: AATAAGCTTTGGACGAGAGGTGAGCA	57
RAB37(pGL3-HS-4)	NM_001006638.3	F: AATCTCGAGTGTGGCTCATGTCCGAAG<br>R: AATAAGCTTTGGACGAGAGGTGAGCA	57
RAB37(pGL3-HS-5)	NM_001006638.3	F: AATCTCGAGGAACAGCAAGGTCCGAG<br>R: AATAAGCTTTGGACGAGAGGTGAGCA	57
RAB37(pGL3-HS-6)	NM_001006638.3	F: AATCTCGAGGAGCCGGTGTCGTCGAG<br>R: AATAAGCTTTGGACGAGAGGTGAGCA	57
RAB37(pGL3-HS-7)	NM_001006638.3	F: AATCTCGAGCTGAGGGGTCCCGGTCGA<br>R: AATAAGCTTTGGACGAGAGGTGAGCA	57
RAB37(pGL3-HS-8)	NM_001006638.3	F: AATCTCGAGCGCTCTCCTTCGCCTGC<br>R: AATAAGCTTTGGACGAGAGGTGAGCA	57
EBF1(Human, CDS)	NM_001324101.2	F: CCCAAGCTTATGTTTGGGATTCAGGAAAGCA<br>R: CCGCTCGAGTCAGGAGAAGGCCAGTCC	58
E2F1(Human, CDS)	NM_005225.3	F: CCCAAGCTTATGGCCTTGGCCGGGG<br>R: CCGGAATTCTCAGAAATCCAGGGGGGT	60
RAB37(HS-EBF1-1-MUT)	NM_001006638.3	F: ATTCTTTCTTCTACTGAGGGGTCCCGGT<br>R: TAGAAGAAAGAATTCCCGTCGCCCCCTC	60
RAB37 (HS-EBF1-2-MUT)	NM_001006638.3	F: CATTTCATTCGGGAGGAGCCTGAGG<br>R: GAATGAAATGCGCCCCCTCGACGAC	59
RAB37 (HS-EBF1-1/2-MUT)	NM_001006638.3	F: CATTTCATTCTTTCTTAGCCTGAGGGGTCCC<br>R: AAGAAAGAATGAAATGCGCCCCCTCGACGAC	59
RAB37 (HS-E2F1-MUT)	NM_001006638.3	F: TTATAATAATGCTGTCGCGGTGCG<br>R: TTATTATAACGAGCCCCGGGCCGT	60
**Mouse**			
Rab37(pGL3-MS-1)	NM_021411.4	F: AATACGCGTTGTGAAGAAGGGAAGTTTGG<br>R: AATCTCGAGGAAGCAAGCGAGGGAGAG	57
Rab37 (pGL3-MS-2)	NM_021411.4	F: AATACGCGTTTCAGCATATCTCTTGGGGG<br>R: AATCTCGAGGAAGCAAGCGAGGGAGAG	57
Rab37 (pGL3-MS-3)	NM_021411.4	F: AATACGCGTTCTCCATATCCCCCGTCATC<br>R: AATCTCGAGGAAGCAAGCGAGGGAGAG	57
Rab37 (pGL3-MS-4)	NM_021411.4	F: AATACGCGTGTGTCGGTGAAAAAGTAGGC<br>R: AATCTCGAGGAAGCAAGCGAGGGAGAG	57

Rab37 (pGL3-MS-5)	NM_021411.4	F: AATACGCGTCTAACTTCACCACTGCGAC<br>R: AATCTCGAGGAAGCAAGCGAGGGAGAG	57
Rab37 (pGL3-MS-6)	NM_021411.4	F: AATACGCGTCCGTGCTGTAAGAGCACTAA<br>R: AATCTCGAGGAAGCAAGCGAGGGAGAG	57
Rab37 (pGL3-MS-7)	NM_021411.4	F: AATACGCGTTCTCAGGGGGACTGCCA<br>R: AATCTCGAGGAAGCAAGCGAGGGAGAG	57
Rab37 (pGL3-MS-8)	NM_021411.4	F: AATACGCGTGAGTCTCAGACGTCCTGG<br>R: AATCTCGAGGAAGCAAGCGAGGGAGAG	57
Rab37 (pGL3-MS-9)	NM_021411.4	F: AATACGCGTGGGAGGAGTCTATCAGGGT<br>R: AATCTCGAGGAAGCAAGCGAGGGAGAG	57
Rab37 (pGL3-MS-10)	NM_021411.4	F: AATACGCGTGAGCCGGTTGGTGGATG<br>R: AATCTCGAGGAAGCAAGCGAGGGAGAG	57
Rab37 (pGL3-MS-11)	NM_021411.4	F: AATACGCGTATGGGGAGGGAGGAGTG<br>R: AATCTCGAGGAAGCAAGCGAGGGAGAG	57
Rab37 (pGL3-MS-12)	NM_021411.4	F: AATACGCGTCTAGGGCAGGGCGGTTCC<br>R: AATCTCGAGGAAGCAAGCGAGGGAGAG	57
Egr2(mouse, CDS)	NM_010118.3	F: CCGGAATTCATGATGACCGCCAAGGCC<br>R: CCGCTCGAGTCACGGTGTCCTGGTTCG	58
E2f1(mouse, CDS)	NM_007891.5	F: CCCAAGCTTATGGCCGTAGCCCCC<br>R: CCGGAATTCTCAGAAATCCAGAGGGGT	58
Rab37 (MS-EGR2-MUT)	NM_021411.4	F: CTTCTGTTTATTCTAAGCTGGCAGGGCGGTTCCT<br>R: AGCTTAGAATAAACAGAAGCCCTCCCCATCCACC	57
Rab37 (MS-E2F1-MUT)	NM_021411.4	F: TTCGTTATTTCATTCTTGAGGAGTCTATCAGG<br>R: AAGAATGAAATAACGAACCTCGACGACGCCAG	58
**Pig**			
RAB37(pGL3-PS-1)	ENSSSCT00025032574.1	F: AATCTCGAGATGCACCCACCTTGTTCC<br>R: AATAAGCTTCCCTGGACGAGAGGTGAG	58
RAB37(pGL3-PS-2)	ENSSSCT00025032574.1	F: AATCTCGAGTGGGAGGTGTGGTGGAAG<br>R: AATAAGCTTCCCTGGACGAGAGGTGAG	58
RAB37(pGL3-PS-3)	ENSSSCT00025032574.1	F: AATCTCGAGGCGCCTGTCCTTTCCCA<br>R: AATAAGCTTCCCTGGACGAGAGGTGAG	58
RAB37(pGL3-PS-4)	ENSSSCT00025032574.1	F: AATCTCGAGATGTCCTCAGGCCAGTGT<br>R: AATAAGCTTCCCTGGACGAGAGGTGAG	58
RAB37(pGL3-PS-5)	ENSSSCT00025032574.1	F: AATCTCGAGCAGGTGGCAGAGCGAAG<br>R: AATAAGCTTCCCTGGACGAGAGGTGAG	58
RAB37(pGL3-PS-6)	ENSSSCT00025032574.1	F: AATCTCGAGCGGCCGGGGTGGCGGAG<br>R: AATAAGCTTCCCTGGACGAGAGGTGAG	58
RAB37(pGL3-PS-7)	ENSSSCT00025032574.1	F: AATCTCGAGGAGGGGAGAGGAGTGGG<br>R: AATAAGCTTCCCTGGACGAGAGGTGAG	58
RAB37(pGL3-PS-8)	ENSSSCT00025032574.1	F: AATCTCGAGCTAAGGCGGGGCGGTTC<br>R: AATAAGCTTCCCTGGACGAGAGGTGAG	58
EBF1(Pig, CDS)	ENSSSCT000000403	F: CCCAAGCTTATGTTTGGGATTCAGGAA	57

	59.2	R: CCGCTCGAGTCACATGGGAGGAACAATCA	
EGR2(Pig, CDS)	ENSSSCT00025106300.1	F: CCCAAGCTTATGATGACCGCCAAGGCC<br>R: CCGGAATTCTCAAGGTGTCCGGGTCC	59
RAB37 (PS-EBF1-MUT)	ENSSSCT00025032574.1	F: TCTTTTCTCTTCTTGGGTGGAGCCTAAG<br>R: AGAAGAGAAAAGACGCCACCCCGGCCGG	59
RAB37 (PS-EGR2-MUT)	ENSSSCT00025032574.1	F: CTTCTGTTTGTTCTAAGCCGGCGGGGCGGTTCCT<br>R: GGCTTAGAACAAACAGAAGCTCCCCTCCGCCACC	59
RAB37 (PS-EBF1+EGR2-MUT)	ENSSSCT00025032574.1	F: TTTCTCTTCTGTTTTGGAGCCTAAGGCGG<br>R: AAACAGAAGAGAAACTCCGCCACCCCGGC	61
RAB37 (pGL3-*RAB37*-*a/b/c/d*)	ENSSSCT00025032574.1	F: AATGTCGACTGGGAGGTGTGGTGGAAG<br>R: AATAAGCTTCCCTGGACGAGAGGTGAG	58
RAB37 (RAB37-a/b/c/d-FLAG)	ENSSSCT00025032574.1	F: AATGTCGACTGGGAGGTGTGGTGGAAG<br>R: AATAAGCTTCCCTGGACGAGAGGTGAG	58
RAB37 (RAB37-a/b/c/d-EGFP)	ENSSSCT00025032574.1	F: TGCTCTAGATGGGAGGTGTGGTGGAAG<br>R: GGAATTCCATATGCCCTGGACGAGAGGTGAG	58
